# Neurodevelopmental defects in a mouse model of *O-*GlcNAc transferase intellectual disability

**DOI:** 10.1242/dmm.050671

**Published:** 2024-04-25

**Authors:** Florence Authier, Nina Ondruskova, Andrew T. Ferenbach, Alison D. McNeilly, Daan M. F. van Aalten

**Affiliations:** ^1^ Department of Molecular Biology and Genetics, Aarhus University, 8000 Aarhus, Denmark; ^2^ Department of Paediatrics and Inherited Metabolic Disorders, First Faculty of Medicine, Charles University and General University Hospital in Prague, Prague, 128 08 Praha 2, Czech Republic; ^3^ Division of Systems Medicine, School of Medicine, University of Dundee, Dundee DD1 9SY, UK; ^4^ Division of Cell and Developmental Biology, School of Life Sciences, University of Dundee, Dundee DD1 5EH, UK

**Keywords:** Intellectual disability, *O-*GlcNAcylation, Vertebrate development

## Abstract

The addition of *O*-linked β-*N*-acetylglucosamine (*O-*GlcNAc) to proteins (referred to as *O-*GlcNAcylation) is a modification that is crucial for vertebrate development. *O-*GlcNAcylation is catalyzed by *O-*GlcNAc transferase (OGT) and reversed by *O-*GlcNAcase (OGA). Missense variants of *OGT* have recently been shown to segregate with an X-linked syndromic form of intellectual disability, OGT-linked congenital disorder of glycosylation (OGT-CDG). Although the existence of OGT-CDG suggests that *O-*GlcNAcylation is crucial for neurodevelopment and/or cognitive function, the underlying pathophysiologic mechanisms remain unknown. Here we report a mouse line that carries a catalytically impaired OGT-CDG variant. These mice show altered *O-*GlcNAc homeostasis with decreased global *O-*GlcNAcylation and reduced levels of OGT and OGA in the brain. Phenotypic characterization of the mice revealed lower body weight associated with reduced body fat mass, short stature and microcephaly. This mouse model will serve as an important tool to study genotype-phenotype correlations in OGT-CDG *in vivo* and for the development of possible treatment avenues for this disorder.

## Teaser

**Summary:** Variants of *O-*GlcNAc transferase are associated with intellectual disability. A mouse model carrying an *O-*GlcNAc transferase variant has altered *O-*GlcNAc homeostasis, reduced mass and microcephaly.

## INTRODUCTION

Intellectual disability (ID) represents a heterogeneous group of neurodevelopmental disorders that are predicted to affect 1–3% of the worldwide population (Maulik et al., 2011). ID appears during childhood and is characterized by impaired cognitive function (with IQ below 70) and adaptive behavior (Shaffer, 2005). Frequent comorbidities are autism spectrum disorders (ASDs), attention deficit and hyperactivity disorder (ADHD), and epilepsy (Posada de la Paz et al., 2017).

The addition of *O*-linked β-*N*-acetylglucosamine (*O-*GlcNAc) to serine and threonine residues of proteins (referred to as *O-*GlcNAcylation) is a dynamic and highly conserved posttranslational modification that regulates several cellular processes, including transcription (Lamarre-Vincent and Hsieh-Wilson, 2003; Constable et al., 2017), signaling (Ong et al., 2018) and metabolism (Whelan et al., 2008). Only two enzymes regulate *O-*GlcNAcylation: *O-*GlcNAc transferase (OGT) is responsible for the addition of a single *O-*GlcNAc moiety on substrates (Kreppel and Hart, 1999) and *O-*GlcNAcase (OGA) for its removal (Gao et al., 2001). *O-*GlcNAcylation has been shown to be essential in mammals. *Ogt* deletion leads to impaired embryogenesis and early development (Shafi et al., 2000; O'Donnell et al., 2004), whereas *Oga* is critical for perinatal survival (Keembiyehetty et al., 2015; Stichelen et al., 2017; Muha et al., 2021). Dysregulation of *O-*GlcNAcylation homeostasis has also been associated with pathological conditions such as diabetes, neurodegeneration and cancers (Bond and Hanover, 2013; Balana and Pratt, 2021; Ciraku et al., 2022).

*O-*GlcNAcylation and the two cycling enzymes are abundant in the mammalian brain (Gao et al., 2001; Okuyama and Marshall, 2003; Akimoto et al., 2003), in particular at pre- and post-synaptic compartments, where many proteins important for neuronal structure and synaptic function have been found to be *O-*GlcNAcylated (Vosseller et al., 2006; Khidekel et al., 2004; Lagerlof et al., 2017; Cole and Hart, 2001). Previous work has demonstrated that modulation of *O-*GlcNAcylation affects neuronal processes important for brain function, including synaptic maturation, synaptic function and axon morphology, and learning and memory (Lagerlof et al., 2017; Francisco et al., 2009; Taylor et al., 2014; Yang et al., 2017; Rexach et al., 2012; Wheatley et al., 2019). Several OGT substrates are also ID-related proteins whose functions have been shown to be regulated by *O-*GlcNAcylation. *O-*GlcNAc represses CREB (cyclic AMP response element-binding protein)-dependent transcription that is associated with changes in neurite growth and long-term memory (Rexach et al., 2012). *O-*GlcNAcylation at T306 also blocks interactions between SynGAP (also known as SynGAP1) and PSD-95 (also known as DLG4) leading to inhibition of liquid-liquid phase separation that could affect postsynaptic density condensate formation (Lv et al., 2022). Although these observations collectively suggest an important role for neuronal *O-*GlcNAcylation in cognition, our understanding of how *O-*GlcNAcylation and the cycling enzymes regulate brain function is still limited.

Recently, missense variants of *OGT* have been identified in individuals affected by ID, giving rise to a syndrome named OGT-linked congenital disorder of glycosylation (OGT-CDG) (Pravata et al., 2020). As *OGT* is located on the X chromosome, almost all individuals with OGT-CDG are male, with the exception of a report of monozygotic female twins both harbouring a *de novo* missense variant (Pravata et al., 2019a). OGT-CDG is a clinically heterogenous disorder in which those affected present with ID, developmental delay and very restricted language skills. Individuals with OGT-CDG also commonly present with dysmorphic features including craniofacial characteristics with broad and high forehead, hypertelorism, broad nasal root, full or long philtrum, and clinodactyly. Brain and eye abnormalities are also observed in most cases (Pravata et al., 2020).

OGT is composed of a catalytic domain and an N-terminal tetratricopeptide repeat (TPR) domain consisting of 13.5 TPRs that is responsible for substrate binding and protein-protein interactions (Hart et al., 2011). In addition to installing *O-*GlcNAc on proteins, OGT is involved in the proteolytic processing and activation of host cell factor 1 (HCF1) (Capotosti et al., 2011), which is encoded by a known ID gene (Castro and Quintana, 2020), and possesses non-catalytic functions implicated in cellular proliferation (Levinea et al., 2021). To date, 17 OGT-CDG missense variants have been reported in *OGT*, with missense and exon-skipping variants in the TPR and catalytic domains giving rise to similar clinical features. This suggests that there are common mechanisms affected in these OGT-CDG variants; these mechanisms remain unknown as no vertebrate models are currently available to dissect them. Investigating the function of OGT in neurodevelopment and the brain has been hampered by mouse lethality caused by global loss of *Ogt* (Shafi et al., 2000; O'Donnell et al., 2004). Although several brain-cell-specific *Ogt* knockout (KO) mouse models also lead to postnatal lethality, these have highlighted the role of OGT in neurodevelopment, neuronal survival and structure (O'Donnell et al., 2004; Wheatley et al., 2019; Shao et al., 2022; Cheng et al., 2020; Wang et al., 2016). Modeling OGT-CDG *in vivo* will help to more precisely understand how OGT regulates processes that are essential for neurodevelopment and brain function.

Here, we report the use of a CRISPR/Cas9 genome editing approach to generate a mouse model carrying a catalytically impaired OGT-CDG variant, C921Y. Unlike previous *Ogt* KO models, mice carrying the C921Y variant of OGT (OGT^C921Y^) are viable, allowing the phenotypic characterization of the animals. Loss of OGT catalytic activity leads to impaired *O-*GlcNAcylation homeostasis in the brain, changes in body size and mass, and microcephaly in OGT^C921Y^ mice.

## RESULTS

### OGT^C921Y^ mutant mice are viable

**Fig. 1.**
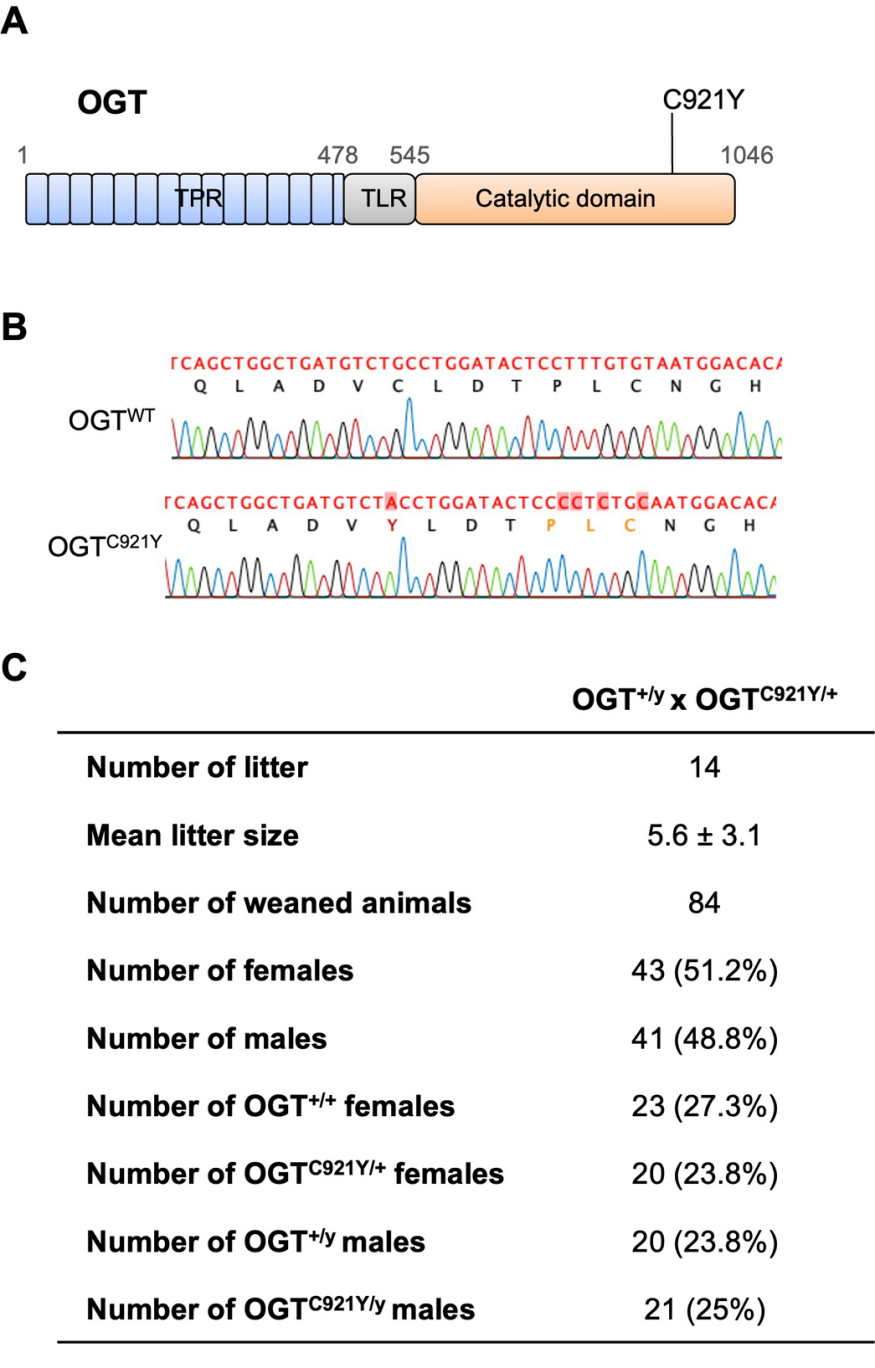
**Genome editing to introduce the OGT^C921Y^ mutation leads to viable mice.** (A) Schematic of the OGT protein with position of the OGT^C921Y^ variant marked. TPR (blue), TPR-like (TLR; gray) and catalytic (orange) domains are represented. (B) Sequencing of genomic DNA of male OGT^WT^ and male OGT^C921Y^ mice confirms the presence of the C921Y point mutation in the transgenic animals. DNA sequence chromatograms for a representative OGT^WT^ and OGT^C921Y^ animal are shown alongside the corresponding DNA and amino acid sequences. The G-to-A point mutation for the C921Y variant is marked in red, as are four silent mutations intended to eliminate gRNA recognition sequences. (C) Table showing numbers and percentages of female (OGT^+/+^), male (OGT^+/y^), female *Ogt^C921Y/+^* (OGT^C921Y/+^) and male *Ogt^C921Y/y^* (OGT^C921Y/y^) animals generated from breeding pairs of female *Ogt^C921Y/+^* and male *Ogt*^+/y^ mice. Litter size is shown as mean±s.d.

To dissect the effects of OGT-CDG variants *in vivo*, we used a CRISPR/Cas9 genome editing approach in mice to introduce the missense mutation C921Y (Omelková et al., 2023), which has been reported previously and is located in the OGT catalytic domain (Fig. 1A). Briefly, zygotes at 0.5 days post-coitum (dpc) were injected with editing reagents and transferred into pseudo-pregnant female mice. DNA from offspring was genotyped and sequenced to confirm the presence of the OGT^C921Y^ mutation (Fig. 1B). As *Ogt* is essential for embryogenesis (Shafi et al., 2000; O'Donnell et al., 2004), we first assessed the viability and mendelian inheritance distribution of offspring generated from heterozygous *Ogt^C921Y/+^* females crossed to wild-type (WT) males (*Ogt^+/y^*) on the same C57BL/6J genetic background. This generated 48.8% male and 51.2% female pups. These observations suggest no sex-related lethality in the OGT^C921Y^ mouse line. DNA from offspring was then genotyped and sequenced, revealing that both heterozygous female and hemizygous male offspring could be obtained for this line. We then analyzed the mendelian inheritance distribution of WT (*Ogt^+/+^* and *Ogt^+/y^*; expected 50%), heterozygous *Ogt^C921Y/+^* (expected 25%) and hemizygous *Ogt^C921Y/y^* (expected 25%) in animals arising from the crosses. We found that the crosses generated 27.3% WT female, 23.8% *Ogt^C921Y/+^* female, 23.8% WT male and 25% *Ogt^C921Y/y^* male offspring at the expected mendelian ratio (*P*=0.29, χ-squared test; *n*=84) (Fig. 1C). We also investigated the fertility of male hemizygotes carrying the OGT^C921Y^ mutation, as some individuals affected by OGT-CDG exhibit genital abnormalities (Pravata et al., 2020). We successfully obtained WT and heterozygous *Ogt^C921Y/+^* animals from WT female and hemizygous *Ogt^C921Y/y^* male breeding pairs, indicating that fertility is not affected in OGT^C921Y^ mice. The gene encoding OGT is located on the X chromosome in both human and mouse, and as almost all individuals with OGT-CDG are male, only hemizygous *Ogt^C921Y/y^* (hereafter referred to as OGT^C921Y^) and WT male animals were used for the following experiments. Taken together, these results show that, contrary to *Ogt* KO models, animals carrying the OGT^C921Y^ mutation in the catalytic domain of OGT are viable.

### The OGT^C921Y^ mutation is linked to changes in brain *O-*GlcNAc homeostasis

**Fig. 2.**
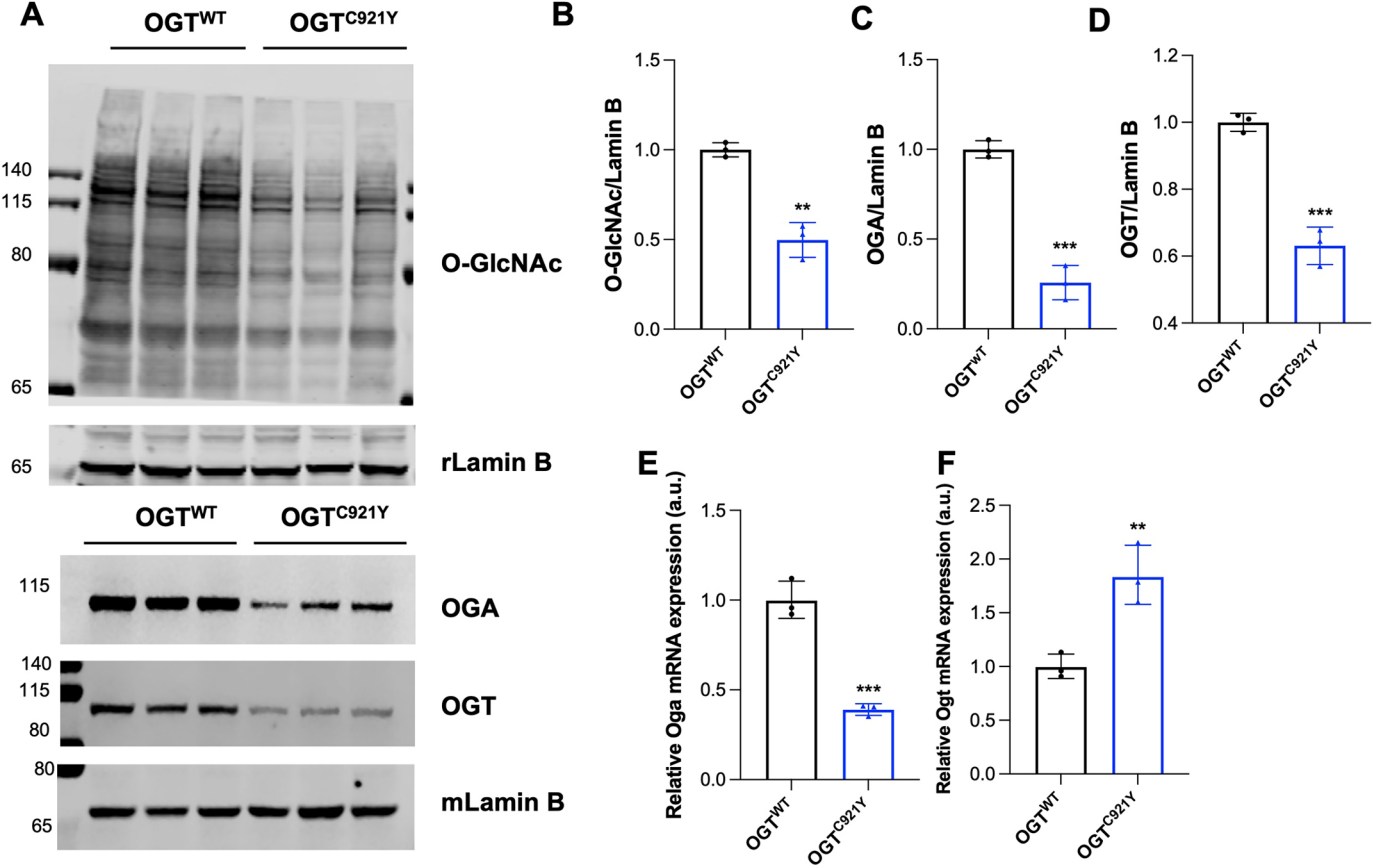
**OGT^C921Y^ mutation causes changes in brain *O-*GlcNAc homeostasis.** (A) Western blot analysis of *O-*GlcNAc, OGA and OGT levels in adult brain of OGT^WT^ and OGT^C921Y^ male mice. Anti-lamin B antibodies raised in rabbit (rLamin B) or mouse (mLamin B) were used as loading controls. Each lane represents independent biological replicates. Molecular masses of markers are shown in kDa. (B) Quantification of total *O-*GlcNAcylated proteins from the western blot shown in A. (C) Quantification of OGA protein levels from the western blot shown in A. (D) Quantification of OGT protein levels from the western blot shown in A. (E) Quantification of *Oga* mRNA levels in whole adult brain of OGT^WT^ and OGT^C921Y^ male mice by RT-PCR. (F) Quantification of *Ogt* mRNA levels in whole adult brain of OGT^WT^ and OGT^C921Y^ male mice by RT-PCR. Protein and mRNA levels are normalized to the mean of the corresponding OGT^WT^ replicate set (a.u., arbitrary units). Data in B-F are represented as mean±s.d., *n*=3 for all genotypes. **P<0.01; ***P<0.001 (two-tailed unpaired *t*-test used).

The OGT^C921Y^ variant affects the catalytic activity of OGT, leading to disruption of *O-*GlcNAcylation homeostasis in stem cells (Omelková et al., 2023). Therefore, we first investigated the levels of OGT, OGA and *O-*GlcNAc levels in whole brains from male OGT^C921Y^ animals (Fig. 2). Global protein *O-*GlcNAc levels were significantly decreased in OGT^C921Y^ males compared to those in WT littermates (Fig. 2A,B). In addition, both OGA (Fig. 2A,C) and OGT (Fig. 2A,D) protein levels were significantly reduced in OGT^C921Y^ animals compared to those in WT mice. To investigate whether the reductions in OGT and OGA protein levels were due to reduced transcription, we performed qPCR analysis on whole-brain tissue extracts from WT and OGT^C921Y^ mice. We observed a reduction in *Oga* mRNA levels in OGT^C921Y^ animals (Fig. 2E), which was accompanied by an increase in *Ogt* mRNA levels (Fig. 2F), suggesting that the reduction in OGT protein levels is not caused by reduced transcription but occurs at the protein level. Taken together, these results show that the OGT^C921Y^ mutation is linked to changes in brain *O-*GlcNAc homeostasis.

### The OGT^C921Y^ mutation affects mouse size, weight and fat mass

**Fig. 3.**
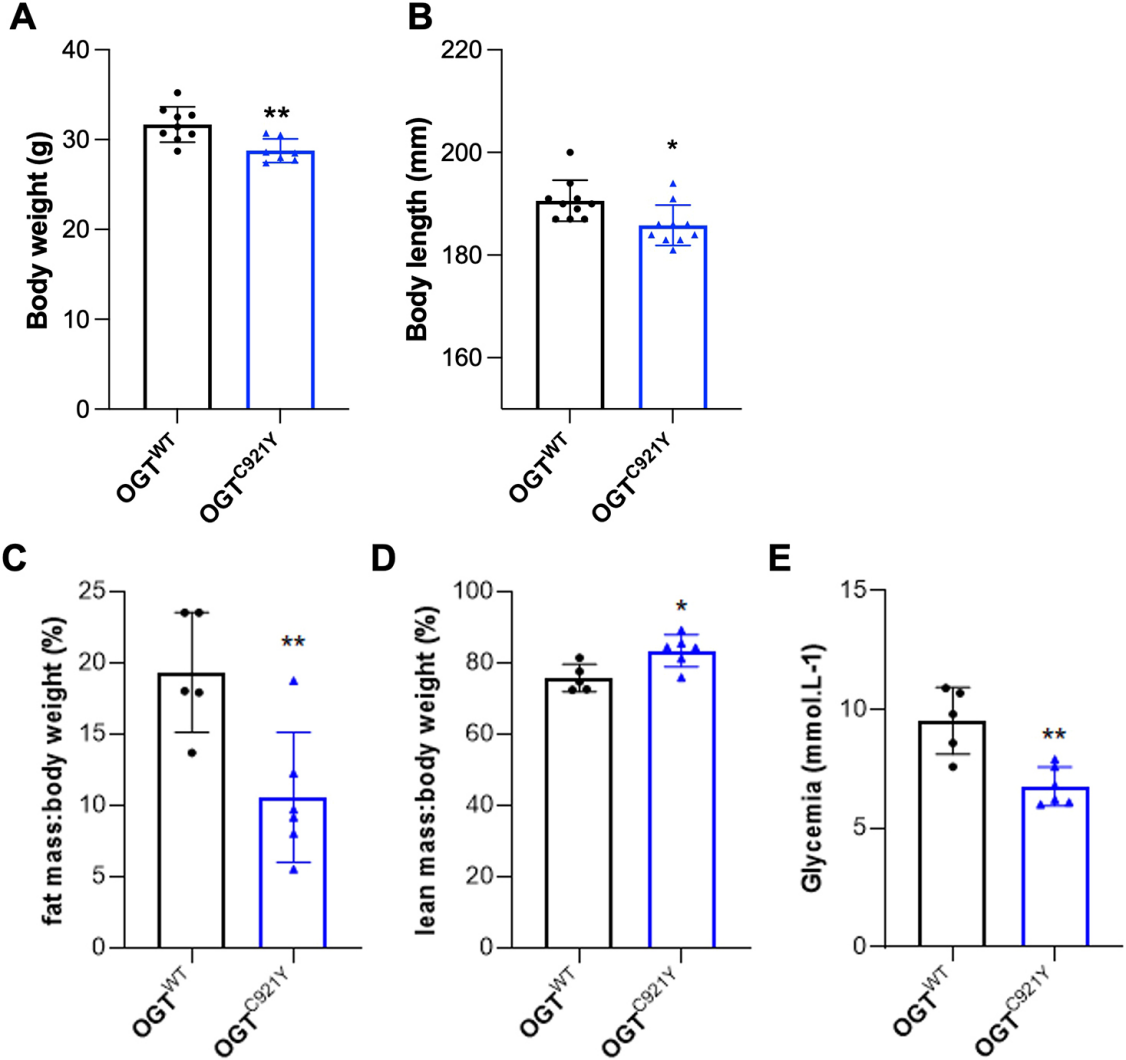
**OGT^C921Y^ mutation leads to changes in mass and size.** (A) Measurement of body weight of 77- to 91-day-old OGT^WT^ (*n*=9) and OGT^C921Y^ (*n*=7) male mice. (B) Measurement of body length (nose-to-tail length) of 77- to 91-day-old OGT^WT^ (*n*=10) and OGT^C921Y^ (*n*=10) male mice. (C) Fat mass:body weight ratio (expressed as a percentage) of 6- to 7-month-old OGT^WT^ (*n*=5) and OGT^C921Y^ (*n*=6) male mice. (D) Lean mass:body weight ratio (expressed as a percentage) of 6- to 7-month-old OGT^WT^ (*n*=5) and OGT^C921Y^ (*n*=6) male mice. (E) Basal glycemia levels of 6- to 7-month-old OGT^WT^ (*n*=5) and OGT^C921Y^ (*n*=6) male mice. Data are represented as mean±s.d. *P<0.05; **P<0.01 (two-tailed unpaired *t*-test used).

Individuals with OGT-CDG commonly present with short stature and low birth weight, and both these features have been observed in people carrying the OGT^C921Y^ variant (Pravata et al., 2020; Omelková et al., 2023). We therefore evaluated morphometric parameters in OGT^C921Y^ mice. We observed a significant decrease in both body weight (Fig. 3A) and nose-to-tail length (Fig. 3B) of OGT^C921Y^ mice compared to that of WT littermates. These quantitative changes were accompanied by a slim appearance of the mutant animals. We next performed EchoMRI imaging to evaluate body composition of the OGT^C921Y^ mice. We observed a significant reduction in body fat mass in OGT^C921Y^ mice compared to that of WT animals (Fig. 3C). The OGT^C921Y^ mice also showed an increase in lean body mass compared to that of WT mice (Fig. 3D). This suggests that the lower body weight observed in the OGT^C921Y^ mice is due to reduced adipose tissue content as well as short stature. In addition, OGT^C921Y^ mice displayed lower levels of glycemia (Fig. 3E) compared to those displayed by WT animals, suggesting that the OGT^C921Y^ mice possess disrupted metabolism. Interestingly, lower glycemia was combined with a significant increase in pancreas weight in OGT^C921Y^ animals compared to that observed for WT animals (Table 1). Taken together, these results suggest that the OGT^C921Y^ mutation affects mouse size, weight and fat mass.

**Table 1.**
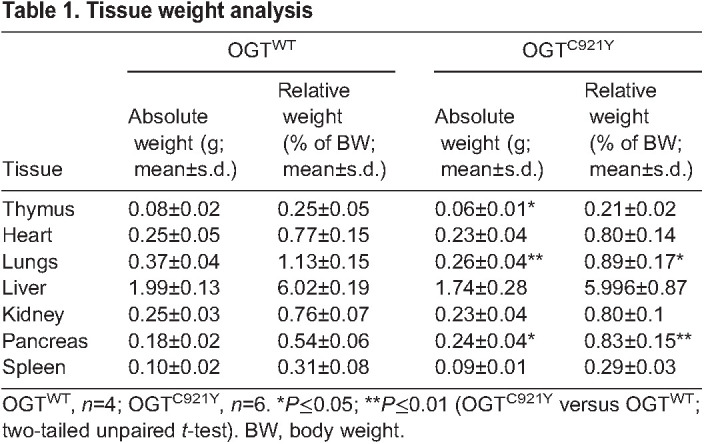
Tissue weight analysis

### Microcephaly and skull deformation in OGT^C921Y^ mice

**Fig. 4.**
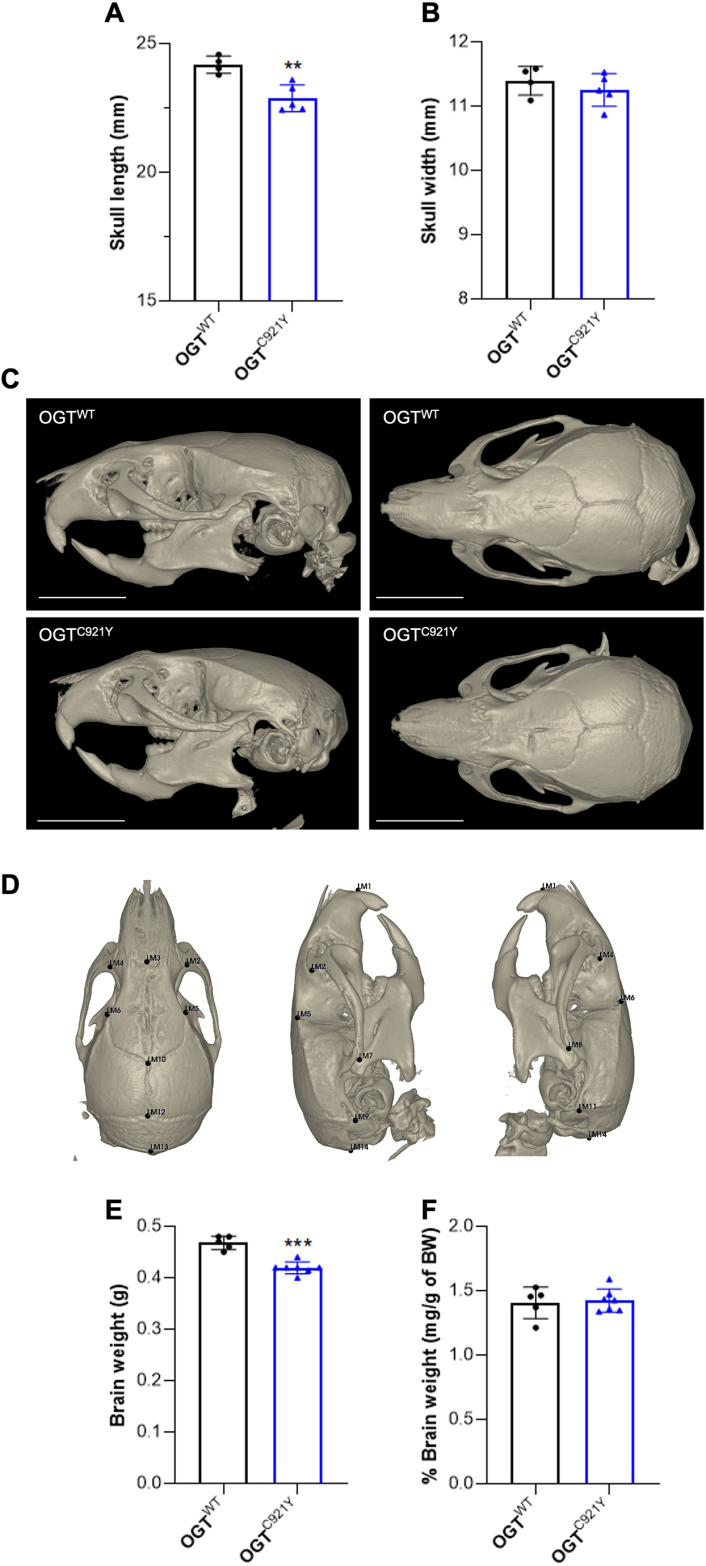
**Microcephaly in OGT-CDG mice.** (A) Measurement of skull length of 80- to 91-day-old OGT^WT^ (*n*=4) and OGT^C921Y^ (*n*=5) male mice. (B) Measurement of skull width of 80- to 91-day-old OGT^WT^ (*n*=4) and OGT^C921Y^ (*n*=5) male mice. (C) Lateral and superior views of representative microCT three-dimensional reconstructions of the skull of 80- to 91-day-old OGT^WT^ and OGT^C921Y^ male animals. Scale bars: 5 mm. (D) Lateral and superior views of a representative microCT three-dimensional reconstruction of a mouse skull presenting 14 landmarks (LM1-LM14) used for Euclidean distance matrix analysis (EDMA). (E) Absolute brain weight of 80- to 91-day-old OGT^WT^ (*n*=5) and OGT^C921Y^ (*n*=7) male mice, as determined using a precision weighing scale. (F) Brain weight:body weight (BW) ratio (expressed as a percentage) of 80- to 91-day-old OGT^WT^ (n=5) and OGT^C921Y^ (*n*=7) male mice. Data in A,B,E and F are represented as mean±s.d. **P<0.01; ***P<0.001 (two-tailed unpaired *t*-test used).

Individuals with OGT-CDG present with craniofacial dysmorphias, which are associated with microcephaly in several cases (Pravata et al., 2020). To first investigate whether the OGT^C921Y^ mutation impacts skull morphology, we acquired measurements and performed microcomputed tomography (microCT) imaging of male OGT^C921Y^ mice and male WT littermates. Using manual measurements, we observed that OGT^C921Y^ mice exhibit a significant reduction in skull length compared to that of the WT mice, whereas skull width was found to be similar between the genotypes (Fig. 4A,B). Analysis of the microCT images revealed that OGT^C921Y^ mice exhibit rounder and smaller skulls compared to those of WT animals (Fig. 4C). Superior and lateral views of the skull microCT images were used for two-dimensional measurement of skull parameters using free landmarks (Fig. 4D). Overall, 72.2% of the linear distances were found to be shortened between 2% and 7% in OGT^C921Y^ animals compared to the distances recorded for WT littermates (Table S1). Along the rostro-caudal axis, 25.6% of the distances were significantly reduced (Table S1), suggesting mild shortening and deformation of the skull in OGT^C921Y^ mice. The reduction in skull size was associated with a significant decrease in the absolute brain weight of OGT^C921Y^ mice relative to the brain weight of WT mice, but this was not accompanied by changes in brain:body weight ratio, owing to the reduced overall body size of the OGT^C921Y^ mice (Fig. 4E,F), suggesting that OGT^C921Y^ mice display a microcephaly phenotype. Taken together, these results suggest that the OGT^C921Y^ mutation leads to mild skull deformation and microcephaly in mice.

## DISCUSSION

We have recently described several variants of *OGT* that are linked to ID (Pravata et al., 2020). To understand the pathophysiology of this disease, we generated a mouse model carrying a catalytically impaired OGT-CDG variant. Although the C921Y mutation causes reduced OGT catalytic activity *in vitro* (Omelková et al., 2023), OGT^C921Y^ animals are viable, allowing the phenotypic characterization of these mice and the investigation of the function of OGT in neurodevelopment. We have shown that the OGT^C921Y^ mutation leads to a reduction in mass and size, altered *O-*GlcNAc homeostasis in the brain of the mice, and microcephaly.

OGT^C921Y^ mice show a reduction in body weight. This was found to be associated with a reduction in body fat mass ratio in OGT^C921Y^ animals compared to that of their WT littermates. Body weight is determined by a balance between food intake and energy expenditure, combining basal metabolism, thermogenesis and physical activity (Speakman, 2013). Compared to WT mice, OGT^C921Y^ mice were found to have lower levels of blood glucose as well as an increase in pancreas size. Similarly, specific *Ogt* deletion in sensory neurons results in reduced body weight and low blood glucose levels in the *Nav1.8-Ogt^KO^* mouse model (Su and Schwarz, 2017). Mice lacking OGT in orexigenic neurons exhibit improved glucose metabolism and are protected from diet-induced obesity (Ruan et al., 2014). Taken together, these observations suggest the involvement of neuronal OGT in controlling basal metabolism that could affect body composition. Interestingly, *Oga^KO/+^* mice exhibit a lean phenotype, similar to our observations of reduced body fat mass in OGT^C921Y^ mice (Yang et al., 2015). *Oga^KO/+^* animals showed increased energy expenditure that was not caused by elevated locomotor activity but by enhanced thermogenesis through an increase in white-to-brown transdifferentiation of adipocytes (Yang et al., 2015). Although possible metabolic defects in OGT^C921Y^ mice remain to be further investigated, these observations suggest that loss of OGT and/or OGA might cause metabolic changes contributing to the lean phenotype observed in OGT^C921Y^ mice.

We observed a reduction in brain size in OGT^C921Y^ mice that was associated with mild skull shortening and deformation. The clinical definition of microcephaly is a reduction in head circumference that implies a decrease in brain size (Zaqout and Kaindl, 2022); we therefore postulate that OGT^C921Y^ mice exhibit a mild microcephaly phenotype. Although microcephaly was not reported in any of the three brothers carrying the OGT^C921Y^ variant (Omelková et al., 2023), it has been observed in individuals carrying other OGT-CDG variants including the OGT L254F, A259T and R284P variants (Gundogdu et al., 2018; Selvan et al., 2018; Willems et al., 2017). The development of the brain depends on several processes, including neurogenesis, neuron migration, and the balance between proliferation and differentiation of neural stem cells. Although *Ogt* has been shown to be important in all these processes (Cheng et al., 2020; Chen et al., 2021; Shen et al., 2021), the mechanisms underlying microcephaly in OGT-CDG remain to be investigated.

Although spontaneous seizure events have been reported for two of the three brothers affected by the OGT^C921Y^ variant (Omelková et al., 2023), we did not observe such seizure events in OGT^C921Y^ mice. Spontaneous seizures are difficult to study as they usually last a few seconds and may occur infrequently. Proper monitoring would require continuous video recording associated with electroencephalograms to detect brain activity in free-moving animals (Gu and Dalton, 2017). Such a setup has been used to observe myoclonic seizure events in a mouse model of Rett syndrome, which is an ID disorder caused by variants of *MECP2* (Shahbazian et al., 2002). Inducible seizures, triggered either chemically or via sensory stimuli (light/sound), have also been demonstrated in mice carrying *Syngap1* and *Fmr1* mutations associated with ID disorders (Clement et al., 2012; Musumeci et al., 2000). Increased sensibility to light and sounds has been reported in an individual affected by the OGT^N648Y^ variant (Pravata et al., 2019b). How OGT^C921Y^ mice might respond to such procedures remains to be explored. This model will also be useful for investigating the effect of OGT-CDG variants on cognition and behavior in mice during early postnatal development and adulthood. Well-established behavioral paradigms can be used to assess developmental delay, locomotion, learning and memory deficits, and social and compulsive behaviors in order to further validate this model as a translational tool to study OGT-CDG.

We have previously discussed potential mechanisms underlying OGT-CDG symptoms (Pravata et al., 2020), and some of these mechanisms have been assessed in our study. Structural analyses of the OGT^C921Y^ mutant protein and other catalytically impaired variants have shown that these mutations affect OGT catalytic activity by disruption of the OGT acceptor binding site, leading to altered substrate binding and/or UDP-GlcNAc binding to OGT, resulting in hypo-*O-*GlcNAcylation of OGT protein substrates *in vitro* (Pravata et al., 2019a; Omelková et al., 2023; Pravata et al., 2019b). This impaired OGT catalytic activity was found to result in a reduction of global *O-*GlcNAc levels in the brain of OGT^C921Y^ mice. Similarly, hypo-*O-*GlcNAcylation has previously been observed in cell and *Drosophila* models carrying OGT-CDG variants (Pravata et al., 2019a,b). However, it is not known whether these mutations will affect all OGT protein substrates or only a subset of them. Given that *O-*GlcNAcylated proteins are particularly abundant in the brain, the loss of *O-*GlcNAcylation on OGT substrates important for proper brain function may contribute to OGT-CDG. Using a filter-based bioinformatics approach combined with structural and clinical data, we have recently predicted the presence of 38 critical *O-*GlcNAc sites across 22 neuronal proteins already reported to be linked to ID and developmental delay (Mitchell et al., 2022). This constitutes a list of potential candidate conveyers for OGT-CDG, and further mechanistic dissection of the OGT-CDG mouse model using a range of ‘omics’-based approaches will help to further understand this disorder. Also consistent with previous findings (Pravata et al., 2019a,b), OGA protein and mRNA levels were decreased in brain of OGT^C921Y^ mice. As OGA has been implicated in intelligence (Savage et al., 2018), and because *Oga* knockdown leads to microcephaly and hypotonia in mice (Stichelen et al., 2017), loss of OGA might also contribute to the OGT-CDG phenotype. To investigate this hypothesis, adeno-associated virus (AAV) vectors or genetic approaches could be used to elevate OGA levels to evaluate whether this could rescue some of the OGT-CDG phenotypes observed in the OGT^C921Y^ mouse model. Unexpectedly, OGT protein levels were also reduced in OGT^C921Y^ mouse brains. This has not been reported previously for cell or invertebrate models of OGT-CDG (Pravata et al., 2020, 2019a). *O-*GlcNAc levels have been demonstrated to regulate *Ogt* transcript levels (Tan et al., 2020). However, our data suggest that the decrease in OGT protein levels is not caused by disrupted transcription. *Ogt* mRNA levels are in fact increased, possibly as a compensatory mechanism to maintain global *O-*GlcNAcylation levels. OGT protein abundance might be disrupted by either protein instability or misfolding leading to proteolytic processing and/or aggregation. Reduction in OGT stability *in vitro* has been previously reported for OGT-CDG variants (Pravata et al., 2019a; Willems et al., 2017; Vaidyanathan et al., 2017; Selvan et al., 2018) but not for the OGT^C921Y^ mutant protein (Omelková et al., 2023; Pravata et al., 2019b). Some OGT-CDG variants cause conformational changes in the protein that could contribute to OGT misfolding (Gundogdu et al., 2018; Pravata et al., 2019b). Although these studies together suggest that OGT-CDG variants could lead to reduced OGT stability, this will require further investigation.

In conclusion, we have successfully generated a mouse model of OGT-CDG that will underpin further investigation of the effects of OGT-CDG variants on brain morphology, brain function and behavior. This model will also be an invaluable starting point to gain insight into OGT-CDG etiology through identification of underlying mechanisms and candidate conveyers of the disease, and will provide a platform for evaluation of potential future treatment strategies.

## MATERIALS AND METHODS

### Generation of OGT^C921Y^ mouse line and animal husbandry

OGT-CDG mice were produced by microinjection under project licence PPL PB0DC8431 at the Central Transgenic Core of Bioresearch and Veterinary Services at the University of Edinburgh, UK. Female C57BL/6J mice (Charles River UK) were superovulated with 5 IU of pregnant mare serum gonadotropin (PMSG; Prospecbio, hoR-272-b) followed by 5 IU of Chorulon (hCG; National Veterinary Services, 804745) 46 h later, and mated overnight with C57BL/6J stud males (Charles River UK). Zygotes were harvested at 0.5 dpc. Editing CRISPR reagents were centrifuged through a Millipore filter (UFC30VV25) and injected into the cytoplasm of zygotes on a Zeiss Axiovert 100 using a Femtojet Xpert (Eppendorf), Transferman 4R (Eppendorf) micromanipulators, Vacutip (Eppendorf) holding pipettes and Femtotip (Eppendorf) injection needles. Injected zygotes were cultured to two-cell stage and then surgically transferred into pseudo-pregnant Crl:CD1(ICR) or Hsd:ICR (CD-1) females (Charles River UK) that had been mated with vasectomized Crl:CD1(ICR) males (Charles River UK) the night before. Genomic DNA from offspring was genotyped and sequenced to confirm the presence of the OGT^C921Y^ variant. Editing CRISR reagents, Cas9 nickase (1081062; Integrated DNA Technologies) and primers were purchased from Integrated DNA Technologies, and sequences are listed in Table S2. Founder OGT-CDG mice were crossed to C57BL/6J WT animals (Charles River UK) for further breeding. Animals were housed in ventilated cages with water and food available *ad libitum* and 12 h/12 h light/dark cycles. All animal studies and breeding were performed on in accordance with the Animal (Scientific Procedures) Act of 1986 for the care and use of laboratory animals. Procedures were carried under UK Home Office Regulation (Personal Project Licence PP8833203) with approval by the Welfare and Ethical Use of Animals Committee of University of Dundee.

### EchoMRI and blood sampling

Body composition data from 6- to 7-month-old male mice was obtained using the EchoMRI 4in1, in line with the protocol provided by the manufacturer (http://www.echomri.com/Body_Composition_4_in_1.aspx). Basal blood glucose levels from tail vein sampling were measured using Bayer Contour glucose meters and strips.

### MicroCT

For MicroCT scanning, 55- to 58-day-old male mice were euthanized and frozen as intact carcasses and defrosted immediately prior to imaging. Carcasses were imaged at 140 kV and 30 μA using a Nikon XTH 225 ST MicroCT scanner at 50 μm resolution. Two-dimensional images were used to generate three-dimensional volumes using 3D slicer (https://www.slicer.org/; Fedorov et al., 2012). Coordinates of 14 landmarks on the skull were recorded from three-dimensional CT images for analysis of the skull morphology. Details of sample genotype were hidden from the researcher performing the landmark analysis.

### Tissue collection and disruption

Brain tissues from 80- to 91-day-old male mice were rapidly dissected, snap frozen in liquid nitrogen and stored at −80°C. Tissues were disrupted in phosphate-buffered saline two times at 5000 rpm for 30 s with 10 s break using a Precellys 24 Touch homogenizer (Bertin Technologies). Homogenates were split in half for further protein and RNA extractions.

### Western blot

Brain homogenates were lysed using 10× RIPA buffer (Cell Signaling Technology), centrifuged at 17,000 ***g*** for 20 min at 4°C, and the protein concentration was determined using a Pierce 660 nm protein assay (Thermo Fisher Scientific). Proteins were separated on precast 4-12% NuPAGE Bis-Tris acrylamide gels (Invitrogen) and transferred to nitrocellulose membrane. Membranes were incubated with primary antibodies in 5% bovine serum albumin in Tris-buffered saline buffer with 0.1% Tween-20 overnight at 4°C. Anti-OGA (1:500 dilution; HPA036141, Sigma), anti-*O-*GlcNAc (RL2) (1:500 dilution; NB300-524, Novus Biologicals), anti-OGT (F-12) (1:1000 dilution; sc-74546, Santa Cruz Biotechnology), mouse anti-lamin B (1:10,000 dilution; 66095-Ig, Proteintech) and rabbit anti-lamin B (1:5000; 12987-1-AP, Proteintech) antibodies were used. Next, the membranes were incubated with IR680- and IR800-labeled secondary antibodies (1:10,000 dilution; 926-32210, 926-32211, 926-68070, 926-68071; LI-COR) at room temperature for 1 h. Blots were imaged using a LI-COR Odyssey infrared imaging system, and signals were quantified using Fiji software (https://fiji.sc/). Results were normalized to the mean of each corresponding OGT^WT^ replicate set and represented as a fold change relative to OGT^WT^.

### qPCR analysis

Total RNA was purified from brain homogenates using an RNAeasy Kit (Qiagen), and then 0.5 to 1 µg of sample RNA was used for reverse transcription with the qScript cDNA Synthesis Kit (Quantabio). Quantitative PCR reactions were performed using the PerfeCTa SYBR Green FastMix for iQ (Quantabio) reagent, in the CFX Connect Real-Time PCR Detection System (Bio-Rad), employing a thermocycle of one cycle at 95°C for 30 s, and then 40 cycles at 95°C for 5 s, 60°C for 15 s and 68°C for 10 s. Data analysis was performed using CFX Manager software (Bio-Rad). Samples were assayed in biological replicates with technical triplicates using the comparative Ct method (Livak and Schmittgen, 2001). The threshold-crossing value was normalized to internal control transcripts (*Gapdh*, *Actb* and *Pgk1*). Primers used for qPCR analysis are listed in Table S3. Results were normalized to the mean of each corresponding OGT^WT^ replicate set and represented as a fold change relative to OGT^WT^.

### Statistics

Statistical analyses were performed with Prism 9. D'Agostino–Pearson, Shapiro–Wilk and Kolmogorov–Smirnov normality tests were performed to verify normality. For data that fulfilled normality requirements, a two-tailed unpaired *t*-test was used for pairwise comparisons of OGT^WT^ and OGT^C921Y^ data. For data sets that did not fulfill normality, a Mann–Whitney test was used for pairwise comparisons of OGT^WT^ and OGT^C921Y^ data. The sample size (*n*) corresponding to the numbers of animals used for each experimental group is indicated in all figure legends.

## Supplementary Material

10.1242/dmm.050671_sup1 Supplementary information

## Data Availability

All relevant data can be found within the article and its supplementary information.
