# Supplementary material for: Neurodevelopmental defects in a mouse model of O-GlcNAc transferase intellectual disability
Source: Dis Model Mech. 2024 Apr 25;17(4):dmm050671. doi: 10.1242/dmm.050671 (PMC11095632; doi:10.1242/dmm.050671)
Supplement: Supplementary information [file dmm-17-050671-s1.pdf]

Table S1. Linear distance ratio of OGT^{C921Y} and OGT^{WT} skulls.

Landmarks	OGT^{C921Y}	*p* value
**1 to 2**	0.995	0.840
**1 to 4**	0.980	0.171
**1 to 3**	1.000	0.953
**1 to 5**	0.980	0.343
**1 to 6**	0.980	0.027*
**1 to 7**	0.976	0.165
**1 to 8**	0.968	0.006**
**1 to 9**	0.966	0.073
**1 to 11**	0.957	0.015*
**1 to 10**	0.986	0.109
**1 to 12**	0.969	0.171
**1 to 13**	0.970	0.076
**1 to 14**	0.963	0.015*
**2 to 3**	1.022	0.314
**3 to 4**	1.045	0.172
**2 to 4**	1.039	0.139
**2 to 5**	0.983	0.130
**4 to 6**	0.991	0.647
**2 to 6**	1.003	0.836
**4 to 5**	1.004	0.863
**2 to 7**	0.950	0.040*
**4 to 8**	0.961	0.084
**2 to 8**	0.991	0.332
**4 to 7**	0.984	0.389
**2 to 9**	0.962	0.024*
**4 to 11**	0.949	0.008**
**2 to 10**	1.000	0.771
**4 to 10**	1.004	0.865
**2 to 11**	0.969	0.024*
**4 to 9**	0.981	0.199

Landmarks	OGT^{C921Y}	*p* value
**2 to 12**	0.965	0.060
**4 to 12**	0.977	0.229
**2 to 13**	0.970	0.067
**4 to 13**	0.977	0.070
**2 to 14**	0.962	0.004**
**4 to 14**	0.965	0.016*
**3 to 5**	0.947	0.038*
**3 to 6**	0.957	0.224
**3 to 7**	0.954	0.050*
**3 to 8**	0.970	0.059
**3 to 9**	0.962	0.010**
**3 to 11**	0.949	0.004**
**3 to 10**	0.969	0.116
**3 to 12**	0.951	0.014*
**3 to 13**	0.961	0.011*
**3 to 14**	0.953	0.002**
**5 to 6**	0.984	0.492
**5 to 7**	0.962	0.152
**6 to 8**	0.987	0.457
**5 to 8**	0.992	0.400
**6 to 7**	0.974	0.196
**5 to 9**	0.982	0.201
**6 to 11**	0.955	0.046*
**5 to 10**	0.991	0.612
**6 to 10**	1.001	0.771
**5 to 11**	0.971	0.034*
**6 to 9**	0,983	0.140
**5 to 12**	0.957	0.056
**6 to 12**	0.971	0.246
**5 to 13**	0.971	0.115
**6 to 13**	0.976	0.170

Landmarks	OGT^{C921Y}	*p* value
**5 to 14**	0.964	0.014*
**6 to 14**	0.963	0.010**
**7 to 8**	0.99	0.338
**7 to 9**	0.942	0.009**
**8 to 11**	0.931	0.072
**7 to 10**	0.972	0.257
**8 to 10**	1.009	0.255
**7 to 11**	0.978	0.164
**8 to 9**	0.99	0.377
**7 to 12**	0.945	0.057
**8 to 12**	0.999	0.960
**7 to 13**	0.956	0.122
**8 to 13**	0.991	0.411
**7 to 14**	0.96	0.004**
**8 to 14**	0.973	0.046*
**9 to 10**	0.996	0.829
**10 to 11**	0.98	0.288
**9 to 11**	0.996	0.782
**9 to 12**	0.985	0.771
**11 to 12**	1.007	0.543
**9 to 13**	0.985	0.603
**11 to 13**	1.014	0.326
**9 to 14**	0.984	0.308
**11 to 14**	0.999	0.954
**10 to 12**	0.936	0.278
**10 to 13**	0.968	0.336
**10 to 14**	0.961	0.051
**12 to 14**	0.967	0.351
**13 to 14**	0.939	0.223

OGT^{WT} vs. OGT^{C921Y} *$p \leq 0.05$; **$p \leq 0.01$; n = 3 (OGT^{WT}) and n = 4 (OGT^{C921Y})

Disease Models & Mechanisms • Supplementary information

Table S2. Sequences of reagents used for introducing the C921Y mutation to *Ogt* gene and genotyping of mice

Guide RNA Left for C921Y	CTCCTGACATGCTCCTCTTT
Guide RNA Right for C921Y	CTGGATACTCCTTTGTGTAA
DNA repair template for C921Y	TATGCACAAAATATGGGCCTTCCCCAGAACCGTATCATTT TCTCACCTGTGGCTCCTAAGGAAGAACACGTCAGGAGA GGTCAGCTGGCTGATGTCTACCTGGATACTCCCCTCTGC AATGGACACACCACAGGGATGGATGTTCTCTGGGCAGG AACACCCATGGTGACTATGCCAGGTTAGTGGCTGATAAA ATC
Genotyping Reverse for C921Y	GAGAGGATGGTGCCAAGTATTCAGGC
Genotyping Forward for C921Y	ATGTGGTTTTAGGGACTTTGTGAGCTC
Sequencing for C921Y	ATGGTGCCAAGTATTCAGGC

Table S3. Primer sequences.

**List of Primers**	**Sequence (5'->3')**
Actb Forward	GATCAAGATCATTGCTCCTCCTG
Actb Reverse	CAGCTCAGTAACAGTCCGCC
Gapdh Forward	ACCCTTAAGAGGGATGCTGC
Gapdh Reverse	GGGACGAGGAAACACTCTCC
Pgk1 Forward	GCTATCTTGGGAGGCGCTAA
Pgk1 Reverse	AAAGGCCATTCCACCACCAA
Ogt Forward	CCCCCTGAGCCCTTCAAAAC
Ogt Reverse	TCGTTGGTTCTGTACTGTCGG
Oga Forward	TGCAGTGGTTAGGGTGTCG
Oga Reverse	AGCAAACGCTGGAACTCTCC
